# Validation of NOVA 27 ultra-processed food screener: adaptation and performance in Ecuador

**DOI:** 10.1017/S1368980025100475

**Published:** 2025-06-04

**Authors:** Wilma B. Freire, Betzabé Tello, Philippe Belmont Guerrón

**Affiliations:** 1 Institute for Research in Health and Nutrition, Universidad San Francisco de Quito, Quito, Ecuador; 2 Center for Research on Health in Latin America (CISeAL), Facultad de Medicina, Pontificia Universidad Católica del Ecuador, Quito, Ecuador

**Keywords:** Validation study, Ultra-processed Foods, NOVA Food Classification, Questionnaire, Diet survey

## Abstract

**Objective:**

This study aimed to adapt and validate the NOVA 27 ultra-processed food (UPF) Screener for use in Ecuador by identifying commonly consumed foods, classifying them using the NOVA system and testing the screener’s validity in an urban sample and a national food survey.

**Design:**

A cross-sectional study was conducted in two phases: screener validation with a convenience sample of 327 adults in Quito through an online questionnaire (2021) and assessment of its applicability using data from the 2012 Ecuadorian National Health and Nutrition Survey (ENSANUT-Ecu). The method, adapted from a similar study in Brazil, compared NOVA UPF scores to the 24 h-Recall (24-HR) automated multiple-pass method, used as the gold standard.

**Setting:**

The study included Quito’s urban population for validation and secondary data from ENSANUT-Ecu.

**Participants:**

Three hundred and twenty-seven adults aged 18–64 from Quito were included in the validation phase, and 3510 adults from the ENSANUT-Ecu dataset were analysed in the secondary analysis.

**Results:**

The screener adaptation identified twenty-seven subgroups of commonly consumed UPF, summarising 90 % of UPF energy intake. Validation results indicated significant agreement between the NOVA-UPF score and UPF intake, with PABAK indices above 0·8 for most socio-demographic groups. Higher NOVA-UPF scores corresponded to increased UPF dietary shares, mirroring patterns observed in the ENSANUT-Ecu dataset.

**Conclusions:**

The adapted NOVA 27 UPF Screener is a valid tool for assessing UPF intake in Ecuador, offering a practical resource for future dietary surveys to monitor and address UPF intake among Ecuadorian adults.

The increasing intake of ultra-processed foods (UPF) has been linked to a rise in non-communicable diseases such as obesity, CVD, diabetes and cancer^(1–3)^. This dietary shift towards high intakes of sugar, fat and salt signifies a transition to poor diet quality, raising public health concerns^(2)^.

UPF fall under the NOVA classification, which categorises foods according to their intended use and degree of processing^(4)^. UPF are industrial formulations made primarily from substances derived from foods, along with additives, containing little or no intact unprocessed food. They often include cosmetic additives, such as colorants, emulsifiers and flavour enhancers, which improve sensory properties, extend shelf life and increase palatability^(4)^. Furthermore, these UPF have been associated with the development and reinforcement of addictive behaviours^(5)^.

The NOVA food classification system categorises foods and beverages into four main groups^(4)^: unprocessed and minimally processed foods, which include natural foods such as fruits, vegetables, legumes, eggs and fresh meats; culinary ingredients, extracted from nature or minimally processed foods, such as oils, butter, sugar and salt, which are used in cooking; processed foods, including pickled vegetables, cheeses and bread made from flour and yeast and UPF, characterised by extensive industrial processing and the presence of ingredients uncommon in traditional culinary preparations. These products often mimic the sensory attributes of their minimally processed counterparts (e.g. flavour and texture), yet their original ingredients are highly modified and designed for rapid consumption^(4)^.

Measuring UPF energy intake typically requires dietary assessment methods such as the 24-HR, which, despite being a standard method, can be costly and time-consuming^(6)^.

In response to the need for an accessible dietary assessment tool, the NOVA UPF Screener tool was developed^(7)^ and validated in adult populations (18 years or older). While this tool has been tested in two other countries, Senegal and Colombia, using similar methodologies, there remains a research gap regarding its adaptation to different cultural contexts, which limits its broader applicability^(6,8)^.

This study aims to adapt and validate the NOVA 27 UPF Screener for Ecuadorian adults (19–64 years old). Older adults were not included, as the ENSANUT-Ecu 2012 database, used as a reference for validation, did not cover this population group.

To achieve this, we applied the screener to a convenience sample and re-analysed national dietary survey data to develop a standardised methodology that accommodates regional dietary patterns, ensuring its effectiveness in capturing UPF intake.

The objectives of this research are twofold:

1. To establish a methodology for adapting the NOVA 27 UPF Screener across different contexts.

2. To validate its utility in monitoring UPF intake in the Ecuadorian adult population.

This study fills a significant knowledge gap, contributing to a more comprehensive understanding of dietary habits.

A versatile UPF assessment tool is essential for public health monitoring. It allows for the rapid assessment of UPF intake across different regions, enabling targeted interventions and evidence-based policy decisions, ultimately promoting healthier dietary practices on a national and global scale.

## Methods

### Design

**Figure 1.**
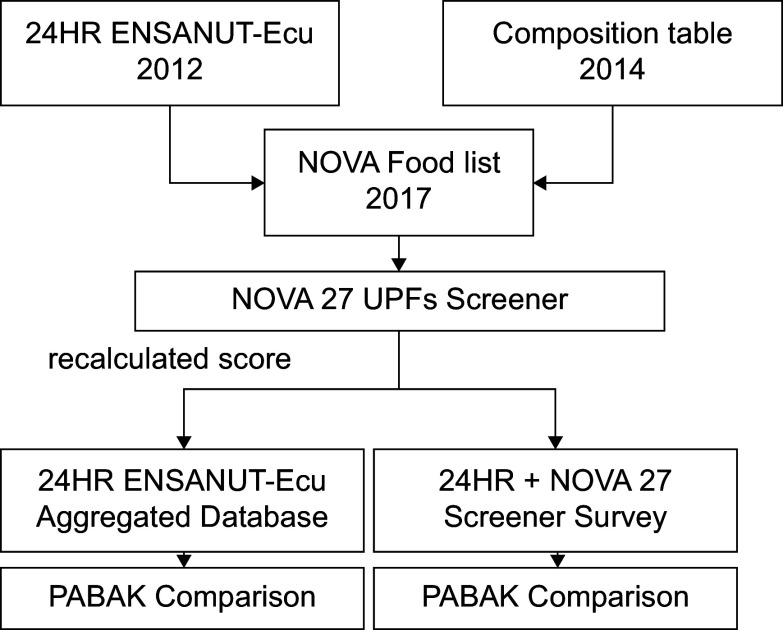
Diagram of data analysis employed in the construction and validation of NOVA 27 UPFs screener.

This cross-sectional study adapted and validated the NOVA 27 UPF Screener for use in Ecuador. The study consisted of two phases: the first was to build a UPF screener based on secondary data and the second focused on validating the screener using a convenience sample and assessing its applicability in a national food consumption survey (see Figure 1).

### Identification of UPF’s most consumed items

To adapt the screener, the first step was to classify foods from the Ecuadorian food composition table with the NOVA classification. This table includes 780 products, along with records of their nutritional composition, and was used in the Ecuadorian National Health and Nutrition Survey 2012 (ENSANUT-Ecu)^(9)^. Each food was assigned to one of the four NOVA classification groups based on its level of processing^(10–12)^. Additional products were incorporated, increasing the total number of food items to 1072^(10)^. Most were ultra-processed products and beverages available in the local market. An online repository containing the methodology and tools used for validation is available at https://revistas.usfq.edu.ec/index.php/bitacora/issue/view/230.

For this study, we refer to ‘categories’ as defined by the NOVA classification and ‘subgroups’ as the food groups created for the screener.

### NOVA 27 UPF screener design

The original NOVA-UPF tool consists of a checklist, developed initially in Brazil with twenty-three subgroups, and has been integrated into the annual ‘VIGITEL survey’ (Brazilian Surveillance System of Risk Factors for Chronic Diseases), conducted via telephone interviews since 2018. The validation study demonstrated its high accuracy in estimating UPF intake as a percentage of total energy intake when compared with the 24-HR^(13)^. The screener has also been included in national surveys, such as the ‘National Adult Health Survey’ and the ‘National School-Age Children Survey’ in Brazil^(13,14)^.

To adapt the NOVA 27 UPF Screener for Ecuador, we used 24-HR data from the ENSANUT-Ecu 2012 survey^(15)^. UPF subgroups were identified, based on their energy contribution, grouping similar items, such as ‘sliced industrial bread’ and ‘toasted industrial bread’ under ‘Sliced or toasted industrial bread.’ The principal UPF for adults aged 18–64 in Quito and other regions were identified (see Table 1). Regional differences were observed, and shared items between regions were retained. The checklist allowed calculating a UPF Score as the sum of UPF subgroups, ranging from 0 to 27.

**Table 1.** Average energy intake in the adult population (18–64): NOVA groups and selected ultra-processed food (UPF) in the national survey ENSANUT-Ecu 2012

Food group	Mean energy intake		% (cal)	% (UPF cal)
	Mean	95 % CI		
NOVA group 1 unprocessed or minimally processed food	1657·5	1614·3, 1700·8	71·33	
NOVA group 2 processed culinary ingredients	238·1	230, 246·2	10·25	
NOVA group 3 processed food	186·1	173·5, 198·7	8·01	
NOVA group 4	242·1	229·7, 254·4	10·42	100·00
Total	2323·8	2247·5, 2400·2	100·00	100·00
Sliced bread or industrial bread	52·1	48·5, 55·8	2·24	21·53
Soda normal or light soft drink	37·8	35·2, 40·4	1·63	15·62
Tea or coffee drink or prepared from powder mix	15	11·6, 18·4	0·65	6·21
Fruit-flavoured drinks or jelly prepared from powder mix	14·9	10·4, 19·4	0·64	6·15
Sausage charcuterie	14·6	11·8, 17·3	0·63	6·02
Sweet cookies with or without filling	13·9	11·9, 15·9	0·60	5·73
Flavored yogurt	8·2	7·1, 9·4	0·35	3·41
Ham, salami or mortadella	6·6	5·7, 7·5	0·28	2·71
Flavored milk	5·5	2·9, 8·2	0·24	2·28
Chips salted snacks	5·4	3·7, 7	0·23	2·21
Fast-food French fries	5·4	3·4, 7·3	0·23	2·21
Chocolate drink or prepared from powder mix	5·1	4·1, 6·1	0·22	2·11
Industrial cake muffin type, branded cake, base cake	5	3·6, 6·5	0·22	2·08
Seasoned meats	4·8	3·1, 6·6	0·21	2·00
Margarine	4·2	3·4, 5	0·18	1·74
Ice cream (neither homemade nor artisanal)	4·1	2·8, 5·5	0·18	1·71
Chocolate and candy	3·4	2·6, 4·2	0·15	1·41
Mayonnaise, Ketchup or mustard	3·1	2·6, 3·6	0·13	1·28
Breakfast cereals	3	1·6, 4·4	0·13	1·25
Frozen pizza or from a fast-food restaurant	1·3	0·6, 2	0·06	0·55
Fruit flavoured drinks, in bottle	1·1	0·5, 1·8	0·05	0·46
Instant pasta or instant soup powder	0·8	0·3, 1·4	0·04	0·35
Empanadas, traditional snacks precooked or frozen	0·8	0, 1·8	0·03	0·33
Coffee creamer and milk powder with additive	0·3	0·1, 0·5	0·01	0·12
Cereal bars	0·2	0, 0·4	0·01	0·09
Salad dressing, bottled	0·2	0·1, 0·3	0·01	0·08
Canned stew	0·1	0, 0·1	0·01	0·02
Total	217	177·6, 256·6	9·34	89·66

The next step involved adapting the 24-HR questionnaire, using the automated multiple-pass food method, to collect consumption data^(16)^.

### Sampling design of ENSANUT-Ecu

The ENSANUT-Ecu 24-HR sampling design involved a subsample drawn from a regionally representative survey. This subsample enabled precise observations at the domain level, covering major regions (coast, highland and Amazon) across both urban and rural settings. In this study, we specifically retained adults from urban highland areas to facilitate population comparisons between the validation study dataset and ENSANUT-Ecu^(9)^.

### Data collection

After adapting the screener and the 24-HR questionnaire, both instruments underwent validation. Initially, two nutritionists reviewed the instruments to ensure the interviewer’s familiarity with the NOVA classification and proficiency in conducting online interviews. The instruments were then tested with a group of ten adults aged 18 and older to refine the questions and ensure comprehension and familiarity with the UPF products. The NOVA classification usage was standardised through pre-classified food exercises.

The sample size was determined based on the expected concordance between the measurements. Based on the literature, a sample of 300 observations is sufficient to detect correlations between methods, though it does not allow stratified analyses by socio-demographic strata. However, comparisons could be made when the Prevalence-Adjusted Bias-Adjusted Kappa (PABAK) agreement is above 0·6^(17)^.

In this study, we included a convenient sample of 327 adults (18–64 years), randomly selected from a telephonic directory. This group was chosen as an initial target, given the potential for self-administered in future surveys. Before participation, individuals received an introductory call explaining the study’s purpose and confirming consent to use two tools for evaluating UPF intake. Upon agreement, a time and location for the online video interview were scheduled. Trained nutritionists conducted data collection, recording both the NOVA-UPF score and a 24-HR of all food items consumed the previous day, along with their respective quantities. On the day of the interview, the screener was applied first over 5 min, followed by the 24-HR automated multiple-pass method, over 30 min. Administering the screener before the 24-HR was designed to elicit more spontaneous responses regarding consumed items, whereas the 24-HR method relies on detailed recall.

Trained interviewers followed a structured protocol to conduct the automated multiple-pass method. A photographic food and beverage atlas was used to facilitate portion estimations, based on previous research^(18)^.

### Statistical analysis

Foods were initially classified into NOVA categories during the interviews. Next, classification were reviewed, portion codes were converted into grams and energy intake was calculated for each NOVA group.

In the secondary data analysis, using the ENSANUT-Ecu 24-HR dataset, the NOVA screener subgroups were identified and reported as consumed the previous day. To prevent duplication, repeated entries were removed for each individual. This approach simulated the completion of the screener in a broader population. In both validations, the present study and ENSANUT-Ecu dataset, the distribution of dietary UPF shares and NOVA-UPF score were calculated using quintile approximations (see Figure 1).

To assess instrument validity, we compared quintiles of NOVA-UPF scores *v*. quintiles of UPF energy intake using the PABAK agreement index. A quadratic weight correction was applied to adjust for subgroup imbalance. Since not all subgroups contribute equally to the agreement, we considered that the difference between the first and second subgroups was less significant than between the second and third subgroups, justifying the use of quadratic weight correction.

Using the national survey data, we examined UPF consumption across different socio-demographic factors, including ethnicity, region, area, and economic well-being index. This latter was developed using principal component factor analysis, incorporating forty-two variables related to housing and household equipment characteristics.

## Results

### NOVA screener adaptation

This initial classification of food items was refined through on-site observation resulting in twenty-seven subgroups (see supplemental material in the online repository mentioned above). These subgroups were categorised into beverages (8), foods (12) and snacks (7). As shown in Table 1, the twenty-seven subgroups accounted for 90 % of UPF energy intake.

The six subgroups with the highest energy contributions represented more than 50 % of total UPF energy intake (see totals in Table 1). Adding more categories provides a wider perspective on the diversity of UPF consumption and can assist in tracking changes over time.

### Population socio-demographic characteristics

Table 2 presents the socio-demographic characteristics of the ENSANUT-Ecu dataset and the validation study sample. In both datasets, the mean age was 33 years, and the proportion of women slightly exceeded that of men.

**Table 2.** Population socio-demographic characteristics in the survey phase (validation study) and national dataset (ENSANUT-Ecu)

Variable	Value	ENSANUT-Ecu		Validation. Study	
		*n*	%	*n*	%
Age	18–24	770	22	58	18
	25–34	1139	32	143	44
	35 or more	1601	46	126	38
Sex	Female	2310	66	179	55
	Male	1200	34	148	45
Education	None	32	1		
	Primary	895	25	9	3
	Secondary	1581	45	75	23
	University	1002	29	243	74
Total		3510	100	327	100

The observed differences indicate that the convenience sample exhibited a more homogeneous distribution, with participants having higher education levels and a younger demographic profile. In total, 327 adults participated in the Quito validation study, while 3510 adults were included in the ENSANUT-Ecu dataset.

### Most consumed subgroups across food groups

**Figure 2.**
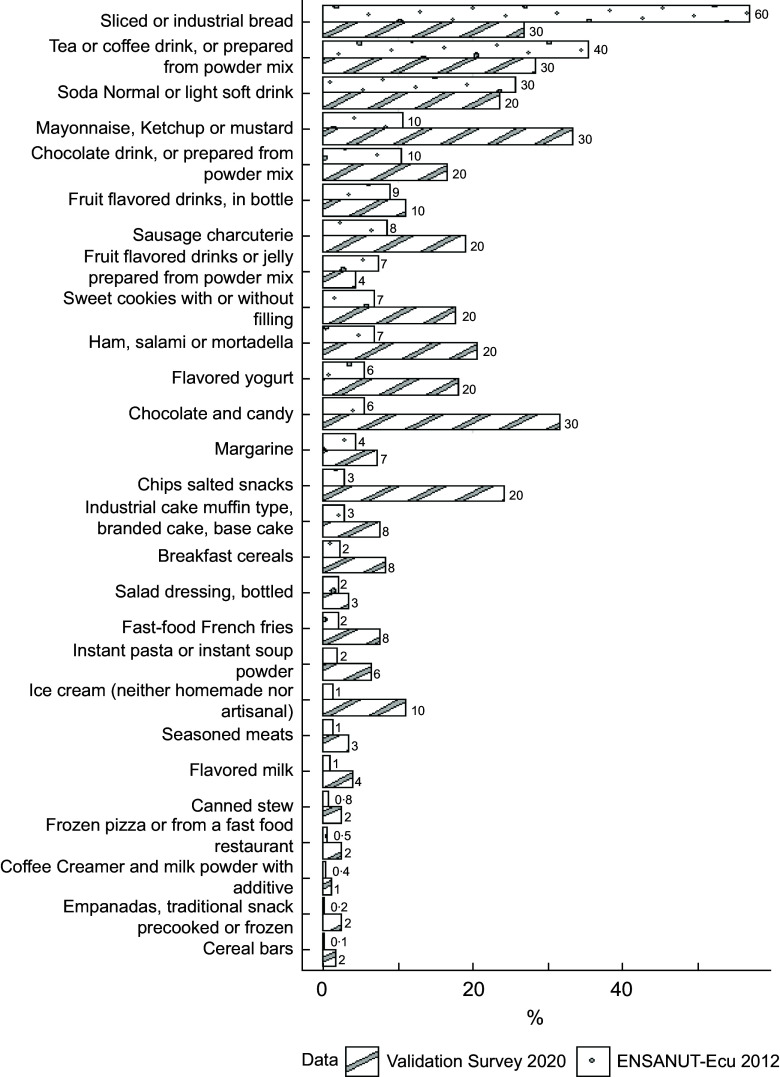
Proportion (%) of consumption on the day before of the food items included in the NOVA 24 h screener for adults (18–64 years old): validation study (Quito) and ENSANUT-Ecu (urban highland).

Figure 2 illustrates the percentage of consumption for each UPF subgroup. The responses from the NOVA 27 UPF Screener were consistent with the 24-HR data from ENSANUT-Ecu, bars with dots indicate ENSANUT-Ecu data, while stripped bars represent the validation data of the study.

In the national dataset, less variation was observed between groups, with the first three subgroups being consumed by over 25 % of the population. In contrast, the validation study sample exhibited greater diversity in UPF consumption.

### Score distribution

**Figure 3.**
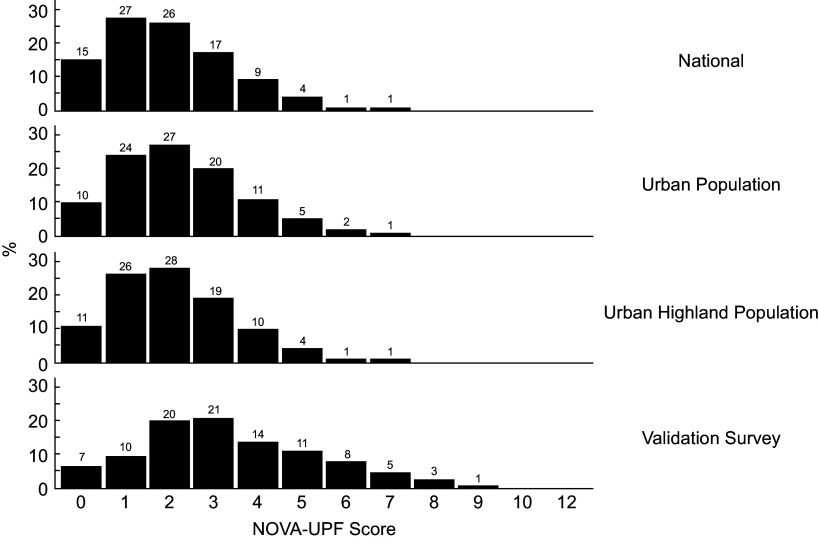
NOVA-UPF score distribution in the validation study and national dataset.

The NOVA-UPF score distribution across the validation study and ENSANUT-Ecu datasets is displayed in Figure 3. In both datasets, scores remained below 13 points, with over 80 % of responses concentrated within the first five points.

Although the score distribution in the first three domains (National, Urban, Urban Highland) from 2012 was relatively similar (0–1–2 scores comprising 68 %, 61 %, and 65 %, respectively), the 2021 validation study showed only 37 % within these ranges. This broader distribution suggests shifts in UPF consumption patterns, reflecting greater diversity and variability between the two periods.

### Mean dietary share

Table 3 shows the mean dietary share of UPF according to the NOVA-UPF score. In both surveys, the increasing score is associated with a significantly increasing contribution of UPF to the total energy intake. In the bottom of Table 3, the score is distributed in five groups, from 0–1 to 5 and more.

**Table 3.** Mean dietary share of ultra-processed food (UPF), adult population (18–64 years old) in the validation study and ENSANUT-Ecu dataset (urban highland)

NOVA-UPF Score	ENSANUT-Ecu				Validation study			
	*n*	%	UPF % of total energy	95 % CI	*n*	%	UPF % of total energy	95 % CI
0	379	10·8	0	0, 0	22	6·7	0·5	0, 1·3
1	893	25·4	5·3	4·8, 5·9***	34	10·4	7·9	4·6, 11·3*
2	993	28·3	11·2	10·5, 11·9***	64	19·6	11·9	9, 14·9***
3	678	19·3	15·4	14·5, 16·2***	70	21·4	20·6	16·6, 24·6***
4	339	9·7	19·5	18·2, 20·9***	45	13·8	26·3	20·8, 31·8***
5	147	4·2	25·7	23·2, 28·1***	36	11	34·4	28·2, 40·6***
6	48	1·4	28·1	23·7, 32·6***	25	7·6	37·4	29·3, 45·4***
7	24	0·7	35·4	28, 42·8***	15	4·6	42·2	29·9, 54·5***
8	5	0·1	42·3	27·1, 57·5***	9	2·8	42·1	25·2, 59***
9	3	0·1	39·4	21·8, 57***	5	1·5	33·3	19, 47·6***
10	1	0	30·7	-, -***	1	0·3	42·4	-, -***
12					1	0·3	70·5	-, -***
0–1	1272	36·2	3·7	3·3, 4·1***	56	17·1	5	2·8, 7·3**
2	993	28·3	11·2	10·5, 11·9***	64	19·6	11·9	9, 14·9**
3	678	19·3	15·4	14·5, 16·2***	70	21·4	20·6	16·6, 24·6***
4	339	9·7	19·5	18·2, 20·9***	45	13·8	26·3	20·8, 31·8***
5+	228	6·5	27·8	25·7, 29·8***	92	28·1	37·6	33·6, 41·7***

*P*-value for linear trend: ***< 0·001; **< 0·01; *< 0·05.

Scores ranged from 0 to 12, with most results ranging from 0 to 5 in both datasets. Although population groups were sampled with distinct objectives and strategies, for a specific NOVA-UPF Score, the energy contribution from UPF is slightly higher in the 2021 validation sample compared to the ENSANUT-Ecu 2012 survey.

### Prevalence-Adjusted Bias-Adjusted Kappa index agreement

Distribution according to the fifths of the dietary share of UPF and approximate fifths of the NOVA-UPF score for the consumption of UPF were compared using the PABAK index. Strength of agreement is considered poor for PABAK values under 0·2, fair to moderate for values ranging from 0·21 to 0·60, and good for values between 0·61 and 0·80, and values above 0·81 considered as very good agreement.

**Figure 4.**
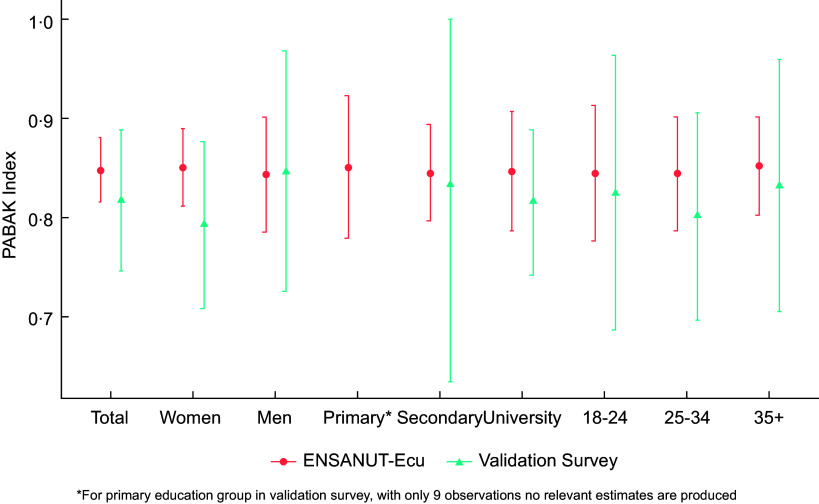
Agreement assessment using PABAK Index for the adult population (18–64 years old) in the validation study (Quito) and ENSANUT-Ecu dataset (Urban Highland) for different socio-demographic groups.

Figure 4 displays the PABAK Index estimates (95 % CI) for socio-demographic groups, comparing the ENSANUT-Ecu dataset with the validation study. We observed strong concordance between NOVA-UPF scores and the percentage of energy intake derived from UPF, with PABAK scores exceeding 0·8 in most demographic subgroups.

The validation study yielded a PABAK of 0·82 (95 % CI: 0·74, 0·88) for the NOVA-UPF score, while the national dataset showed PABAK of 0·85 (95 % CI: 0·82, 0·89). The results demonstrated consistent agreement across sex, education levels and age groups, with all point estimates exceeding 0·8, except for women in the validation study (PABAK index: 0·79).

## Discussion

The NOVA score for UPF consumption, adapted in this study using a rapid self-report questionnaire (5 min), showed a linear association with UPF energy intake, measured through 24-hour dietary recall. This correlation was consistently observed in both the validation study and the national dataset. The PABAK index across various population subgroups showed good to very good agreement, with values above 0·8 in all cases when using national survey data.

Alongside this study, four other countries have conducted validation research using similar tools: Brazil^(7)^, Colombia^(8)^, Senegal^(6)^ and India^(19)^. In Colombia, findings showed a strong association between high UPF consumption and increased intake of sugar, Na and fat among adolescent females^(8)^. Notably, the UPF items included in the Colombian and Brazilian screeners were almost identical, demonstrating high concordance and a consistent pattern of UPF consumption. Meanwhile, in Senegal, new food items were introduced, but the primary UPF subgroups remained consistent with those identified in Brazil^(6)^. Conversely, the Indian adaptation required substantial modifications due to the distinct nature of traditional dishes, which differ significantly from those in Latin American and Africa^(19)^. These findings underscore the tool’s potential for policy monitoring and interventions aimed at reducing UPF intake.

The growing recognition of food processing level as a key factor in dietary research has reinforced its importance in developing nutrition policies and public health programs. Current research provides compelling evidence linking UPF consumption to increased risks of obesity, cardiovascular diseases, mental disorders and mortality^(3)^.

Consequently, it is crucial to develop easily applicable tools that provide reliable dietary data to estimate UPF intake and its impact on health outcomes.

### Scope

When national 24-HR dietary data is available, field validation can be replaced by score validation using the 24HR data, provided it includes the NOVA classification. However, many dietary surveys lack NOVA classification, necessitating a post hoc reclassification approach. Evidence from various countries, including Ecuador, suggests that incorporating specific food item characteristics (such as the brand name, preparation location and consumption setting) enables this reclassification. This strategy enhances the feasibility of integrating NOVA classification into existing dietary surveys.

However, some classification challenges remain. Differentiating between industrially processed and homemade versions of similar foods may lead to misclassification errors, particularly when auxiliary information is lacking. Additionally, relying on self-reported dietary intake assumes perfect recall, which is often not the case in practice.

To ensure correct identification of UPF in 24-hour surveys, surveyors must be thoroughly trained in NOVA classification. This study demonstrates that online validation is feasible and cost-effective, with results comparable to face-to-face validation. Additionally, integrating food atlases into online surveys could further enhance product identification accuracy^(20)^.

Using national survey data, we observed regional variations in UPF consumption. Despite these regional differences, the strong agreement between NOVA-UPF score and dietary intake highlights the tool’s robustness across socio-demographic groups.

Similar to poverty measurement, dietary methodologies based on distribution patterns could benefit from a multidimensional approach, as is feasible with NOVA 24-HR data^(21,22)^. This approach could support policymakers in designing effective malnutrition reduction strategies. As suggested by Costa, the NOVA-UPF screener provides an efficient way to monitor UPF consumption trends, making it easily adaptable for national surveys^(22)^.

Integrating a UPF screener into national well-being and health surveys could facilitate regular updates and a deeper understanding of UPF determinants. For household surveys – including 24-hour dietary recall (24HR) – it is essential to incorporate NOVA classification. This would enable the collection of standardised dietary data across multi-purpose surveys and well-being household surveys.

### Limitations

The primary limitation of this study is the use of secondary data from 2012, which may not fully reflect current UPF consumption patterns. However, the high level of agreement observed in the validation process supports the tool’s continued relevance.

A notable limitation of the validation study was the use of a convenience sample, which aimed to replicate observed correlations from other studies. Despite this, the results demonstrated high consistency when compared to national survey data, particularly regarding PABAK agreement indices across different populations.

Estimating portion sizes remains a challenge. FFQ, for instance, are susceptible to self-selection bias, which can distort dietary intake estimates. Alternative dietary assessment methods are needed to address these issues^(17)^. The 24-HR dietary recall, despite being highly comprehensive, is costly to implement at a national scale. Large samples help account for variation due to race/ethnicity, education and dietary interventions, but continuous follow-up studies remain prohibitively expensive. Nutrition screening tools that produce comparable metrics have demonstrated reliable results in the previous studies but may overestimate moderate-risks population^(17)^. Similarly, a FFQ developed for Ecuador exhibited good validity and reliability when adjusted for energy intake^(23)^.

### Concluding remarks

Changes in the consumption of UPF evolve with the agro-industrial offer. Between ENSANUT-Ecu 2012 and the 2021 validation study, UPF have become more prevalent, increasing their total energy contribution to the Ecuadorian diet. This trend underscores the need for continued monitoring and further investigation into consumption patterns.

The UPF industry has actively introduced products that mimic traditional foods but are industrially manufactured, often containing artificial additives aimed at fostering habitual consumption^(24)^. This trend suggests a homogenisation of UPF dietary patterns, potentially leading to standardised consumption behaviours. Furthermore, the UPF industry employs aggressive marketing strategies, targeting consumers through advertising, packaging innovations and strategic product placement to increase UPF consumption. These tactics contribute to the normalisation of UPFs in daily diets, further reinforcing habitual intake and shaping consumer preferences from an early age.

Further analysis of 24-HR data may help uncover specific consumption trends, particularly among populations with low UPF exposure, in countries experiencing nutritional transition^(8)^. It would also be valuable to assess the substitution of unprocessed and homemade processed foods with UPF, particularly regarding the role of flavour enhancers and colourants in fostering dependency.

This study highlights the effectiveness of the NOVA 27 UPF Screener in promoting transparent and standardised dietary assessments. The tool can be applied both nationally and internationally, facilitating comparative research across countries. Its integration into national surveys could contribute to better policymaking and the development of evidence-based nutritional guidelines aimed at reducing UPF consumption and improving population health.
